# Minimal persistent inflammation in Japanese cedar pollinosis and a prophylactic effect of intranasal corticosteroids

**DOI:** 10.1186/2045-7022-3-S2-P27

**Published:** 2013-07-16

**Authors:** Yasuyuki Noyama, Mitushiro Okano, Misato Hirai, Takenori Haruna, Kazunori Nishizaki

**Affiliations:** 1 Okayama University Graduate School of Medicine, Dentistry and Pharmaceutical Sciences, Okayama, Japan; 2 Okayama University Graduate School of Medicine, Dentistry and Pharmaceutical Sciences, Department of Otolaryngology-Head & Neck Surgery, Okayama, Japan; 3 Okayama Saiseikai General Hospital, Department of Otolaryngology-Head & Neck Surgery, Okayama, Japan

## Background

Low doses of allergen exposure can cause an activation of inflammatory cells in nasal mucosa without an onset of nasal symptoms, called MPI (minimal persistent inflammation). MPI contributes to hyperreactivity and subsequently onset of the full-scale symptom. However, little is known whether MPI is present in JCP (Japanese cedar pollinosis). In addition, a prophylactic effect of intranasal corticosteroids on MPI in JCP has not been investigated.

## Method

We designed a double-blinded, randomized, placebo-controlled, crossover trial. 20 patients with JCP and without perennial allergic rhinitis were enrolled. Nasal provocation test with low dose of allergen (14.7µg/disc) was performed once daily for 3 consecutive days. The levels of ECP and tryptase in nasal discharge were examined. Patients started to receive FFNS (fluticasone furoate nasal spray) or placebo one day before the first nasal provocation test.

## Result

In placebo group, only 25% of patients showed positive response to the provocation test on day 1. However, 75% and 68% of patients showed positive response on day2 and day3, respectively. After the first provocation, the levels of ECP and tryptase were both significantly increased. These levels were not significantly different between the positive and negative responders, and the increase was seen even in the negative responders. Pretreatment with FFNS significantly suppressed the increase of nasal ECP and tryptase.

## Conclusion

These results suggest that MPI characterized by the up-regulation of ECP and tryptase is present in JCP, and a prophylactic treatment with intranasal corticosteroids has a potential for controlling MPI in JCP.

